# Stance Postural Strategies in Patients with Chronic Inflammatory Demyelinating Polyradiculoneuropathy

**DOI:** 10.1371/journal.pone.0151629

**Published:** 2016-03-15

**Authors:** Steno Rinalduzzi, Marco Serafini, Marco Capozza, Neri Accornero, Paolo Missori, Carlo Trompetto, Francesco Fattapposta, Antonio Currà

**Affiliations:** 1 Neurology and Neurophysiopathology Unit, Sandro Pertini Hospital, Rome, Italy; 2 Department of Neurology and Psychiatry, Sapienza University of Rome, Rome, Italy; 3 Neurosurgery Unit, Policlinico Umberto I, Department of Neurology and Psychiatry, Sapienza University of Rome, Rome, Italy; 4 Institute of Neurology, Department of Neurosciences, Ophthalmology and Genetics, University of Genova, Genova, Italy; 5 Academic Neurology Unit, A. Fiorini Hospital, Department of Medical-Surgical Sciences and Biotechnologies, Sapienza University of Rome, Terracina (LT), Italy; University Medical Center Groningen UMCG, NETHERLANDS

## Abstract

**Introduction:**

Polyneuropathy leads to postural instability and an increased risk of falling. We investigated how impaired motor impairment and proprioceptive input due to neuropathy influences postural strategies.

**Methods:**

Platformless bisegmental posturography data were recorded in healthy subjects and patients with chronic inflammatory demyelinating polyradiculoneuropathy (CIDP). Each subject stood on the floor, wore a head and a hip electromagnetic tracker. Sway amplitude and velocity were recorded and the mean direction difference (MDD) in the velocity vector between trackers was calculated as a flexibility index.

**Results:**

Head and hip postural sway increased more in patients with CIDP than in healthy controls. MDD values reflecting hip strategies also increased more in patients than in controls. In the eyes closed condition MDD values in healthy subjects decreased but in patients remained unchanged.

**Discussion:**

Sensori-motor impairment changes the balance between postural strategies that patients adopt to maintain upright quiet stance. Motor impairment leads to hip postural strategy overweight (eyes open), and prevents strategy re-balancing when the sensory context predominantly relies on proprioceptive input (eyes closed).

## Introduction

Human postural control can be studied with the single- or multi-link inverted pendulum model [1–7]. This model assumes two strategies (ankle and hip centered), that can either be used by the central nervous system to produce adaptable control over the horizontal position for the center of mass in the sagittal plane. The ankle strategy repositions the center of mass by moving the whole body as a single-link inverted pendulum by producing torque at the ankle, other standing joints (knee, hip, vertebrae, neck) remaining rigid. Conversely, the hip strategy moves the body as a double-link inverted pendulum with counter-phase motion at the ankle and hip, all other standing joints (knee, vertebrae and neck) remaining rigid. The ankle strategy involves early dorsal ankle muscle activation followed by dorsal thigh and trunk muscle activation (for responses to backward translations). These muscle activations produce torque at the support surface. Kinematic analyses therefore show body movement predominantly at the ankle joint, and only small movements at the hip. The hip strategy entails early ventral trunk and thigh muscle activation associated with a relative increase in shear forces at the support surface and little phasic activation in ankle muscles [8, 9]. Kinematic analyses show trunk flexion paired with ankle extension [8, 9]. Ankle and hip strategies are not extremes along a continuum of mixed strategies, rather simultaneously co-existing excitable modes, always present together, one predominating on the other depending on the available sensory information, task or perturbation [10–11]. Almost “pure” ankle or hip strategy is observed in response to specific perturbations. For example, the ankle strategy persists during small perturbations consisting of low-amplitude, low-velocity or low frequency stimuli. With larger perturbations, the hip strategy predominates [10].

Somatosensory input during postural movement control involves muscle sensory organs, such as spindles, and Golgi tendon organs [12–14]. Proprioceptive signals travel to the brain through large peripheral nerve fibers, the fibers stimulated during routine nerve-conduction studies. To interpret complex sensory environments the brain needs to weigh their relative dependence on each of the senses, integrating proprioceptive information with visual and vestibular information. When tested while standing on a firm support base, in a well-lit environment, healthy persons primarily rely on somatosensory and to a lesser extent on vestibular and visual information [15]. When they have to move in a dimly lit garden, sensory information is re-weighted according to the new sensory context. This ability is important for maintaining stability, and individuals with somatosensory impairment from peripheral polyneuropathy find it difficult to re-weigh postural sensory dependence according to changing sensory context, and are therefore at risk of falling [16]. The loss of sensory perception secondary to diabetic distal symmetrical sensory neuropathy markedly deteriorates postural stability, induces larger and faster postural sway than normal and these deficit are greatest when visual or vestibular cues are absent or degraded [17–18].

Another major factor in maintaining balance is ankle strength. Patients with motor impairment related to peripheral polyneuropathy perform weak, slowed, and delayed flexion-extension foot movements, and they have difficulty to recover from lateral perturbations for their inability to develop torque rapidly about the ankle and hip joints, related to the severity of neuropathy [19–22]. Individuals with muscle weakness without concomitant sensory loss also find it difficult to maintain postural control based on proprioceptive input. This relative failure of proprioceptive postural control associated with muscle weakness indicates a functional link between contractile and sensory muscular processes [23].

Patients with peripheral polyneuropathy due to diabetes have postural instability and an increased risk of falling [24–26]. Many studies have tried to describe and quantify postural sway in these patients by using electromyography and posturography [27–31]. In earlier research, we used the bisegmental posturography technique to describe the balance between hip or ankle postural strategies that could differentiate between young and older healthy people [32]. We found that normal elderly humans operated postural control by overweighing ankle strategy, whereas a predominant two-link model (with more balanced hip and ankle strategies) was preferred in young healthy persons. Few reports describe postural strategies during upright stance in polyneuropathy due to diabetes or somatosensory loss due to experimental ischemia [33–35]. Knowing more about postural strategies for neural control of balance to prevent falls may help in developing a functional diagnostic tool for clinical practice or for monitoring balance improvements in patients with a specific peripheral polyneuropathy, such as chronic inflammatory demyelinating polyradiculoneuropathy (CIDP). CIDP is a disabling distal-proximal sensory-motor neuropathy that mainly affects large myelinated fibers causing focal demyelination and axonal damage [36]. Since it is chronic, the eventual compensatory mechanisms adopted to control balance are likely well established. Since it affects both motor and sensory fibers, this neuropathy offers the opportunity to study how postural changes take place when motor impairment summates to sensory loss. Finally, since this clinical condition affects both proximal and distal nerve segments theoretically it may impair activation of ankle vs. hip strategy unequally.

Our aim in this clinical and posturographic investigation was to use the bisegmental posturography technique to investigate the balance between postural strategies adopted for controlling stance under static conditions in patients with impaired somatosensory input and motor function related to CIDP at distal and proximal muscle districts of the lower limbs. To do so, in patients with CIDP and healthy subjects standing on the floor, as a postural variable we measured body sway (velocity and amplitude) and as a kinematic variable reflecting postural strategies (single-segment or double-segment inverted pendulum) we calculated the mean direction difference (MDD) in the velocity vector from the two electromagnetic trackers placed at two body levels, head and hip. In order to assess the visual feedback on postural control we tested the eyes open and eyes closed conditions.

## Materials and Methods

For bisegmental posturography, we used two electromagnetic trackers (Flock of Birds Motion Tracking; Ascension Technology Corporation; Shelburne, Vermont, USA) connected to a computer. Detailed information about this technique has been published elsewhere [32]. Subjects stood upright on the floor with their feet together and without shoes. Each subject wore two trackers: one (T1) placed on the back of the head (inion) and the other (T2) on the back of the hip (at L5), both secured with elastic belts. Body sway was studied by recording the sway from the two trackers during quiet stance, with subjects standing on the floor with feet together, in two different trials, eyes open and eyes closed. The following postural and kinematic variables were analyzed: mean velocity (MV) and mean amplitude (MA) of each sway tracker, and the mean difference of direction (MDD) between T1 and T2, calculated as the mean of the instantaneous angular difference between the velocity vector for the two trackers. MDD values provided information about flexibility in the ankle-hip-head axis. The lower MDD value reflected predominant ankle strategies and the higher MDD value corresponded to hip strategies [32]. Arbitrary units were chosen to obtain a value of 0 for parallel vectors; 0.5 for perpendicular vectors; and 1 for antiparallel vectors. MDD values ranged from 0 to 1. Whereas the system monitored tracker displacement in three-dimensional (3D) space, we considered only the, x, y displacement, z movement (vertical) being relatively minimal.

We recruited for the study 13 patients of both sexes aged 24–67 years (mean: 46.7 years) with CIDP (mean years of disease 3±1.8) (Table 1) and 24 healthy control subjects of both sexes aged 20–67 years (mean: 42 years) with no history of neurologic abnormality.

**Table 1:** Clinical Characteristics of Subjects at the time they participated in the study.

Patient	Age	Sex	Age of onset	NDS-MP	NDS-MD	NDS-S
1	24	M	20	2	12	2
2	28	M	27	4	16	2
3	32	F	31	12	15	8
4	45	F	39	8	17	8
5	47	F	45	4	18	6
6	51	F	45	18	16	16
7	50	M	46	5	16	8
8	60	M	58	4	12	10
9	54	M	51	4	12	12
10	67	M	66	4	11	14
11	62	F	60	12	13	8
12	50	F	47	4	17	2
13	38	M	34	6	13	6

NDS neurological disability score [37]

NDS-MP scores of lower limb proximal muscle disability (3 muscles tested on left and right leg)

NDS-MD scores for lower limb distal muscle disability (3 muscles tested on left and right leg)

(0 = normal strength; 1 = 25% paresis; 2 = 50% paresis; 3 = 75% paresis; 4 = paralysis)

NDS-S scores for lower limb distal sensory disability: tested on both halluces; four tests: touch, pain, vibration, position sense; 0 = normal sensation; 1 = reduced sensation; 2 = no sensation).

Patients with CIDP were recruited from the Neurology department and diagnosed according to clinical findings, nerve conduction study, cerebrospinal fluid examination, and laboratory tests [38–39]. Patients were selected from a larger group, none of whom had other underlying disorders such as diabetes, prolonged alcohol abuse, chronic renal failure. The patients were studied in stabilized clinical condition, when treatment was discontinued. All patients with CIDP had a stepwise progressive disease course. According to the CIDP Disease Activity Status (CDAS) [40] all patients were classified as being in “remission”. All were subjected to posturography in the remitting or stable stage. Each patient was evaluated with the neurological disability score (NDS) [37], electrodiagnostic testing, bisegmental posturographic recording and cerebrospinal fluid examination. Healthy control subjects underwent posturographic recording alone. Electrodiagnostic studies provided neurographic data including sural-nerve sensory conduction velocity and bilateral peroneal and tibial-nerve motor conduction velocity. In all the patients studied, neurographic data showed symmetric sensory and motor fiber impairment (Table 2).

**Table 2:** Mean values from nerve conduction studies in CIDP patients.

	Nerve Stimulated		
	Sural (s)	Peroneal (m)	Tibial (m)
**Latency (ms)**	4.3 ± 0.5 (UNL ≥4.4)^§^	6.9 ± 1.5 (UNL ≥6.5)	8.9 ± 2.3 (UNL ≥5.8)
Amplitude*	4.3 ± 3.0 (LNL ≥6.0)	2.1 ± 0.6 (LNL >2.0)	3.5 ± 1.7 (LNL ≥4.0)
**Velocity (m/s)**	27.6 ± 6.4 (LNL ≥40.0)	26.5 ± 5.0 (LNL ≥44.0)	27.4 ± 4. (LNL ≥41.0)
**F wave latency (ms)**		67.4 ± 4.6 (UNL ≤56.0)^#^	69.1 ± 6.4 (UNL ≤56.0)

s: sensory study; m: motor study; UNL, upper normal limit; LNL, lower normal limit.

*Amplitude: motor studies in mV, sensory studies in μV.

^§^absent in three patients

^#^absent in five patients

Values represent means ± standard deviations.

Neurological examination excluded vestibular impairment. Cerebrospinal fluid examination in all the patients showed moderately increased protein concentration without pleocytosis (<10 cells/mm^3^). All the patients and healthy subjects received detailed information about experimental procedures and provided written informed consent before attending to the study. The study was performed in agreement with the Declaration of Helsinki and approved by the institutional ethical committee of the Department of Neurology and Psychiatry, Sapienza University of Rome.

Posturographic data were expressed as median plus or minus standard error (SE). Neurographic data were expressed as mean plus or minus standard deviation. Wilcoxon test was used to evaluate differences between eyes open and eyes closed conditions whereas Kruscal-Wallis test was used to evaluate all variables differences between healthy subjects and patients with CIDP. Spearman’s test was used to determine possible correlations between postural variables and NDS. P values equal to or less than 0.05 were considered to indicate statistical significance.

A supporting Information file, S1 File (Clinical, neurophysiological and posturographic measures in healthy subjects and patients) is provided containing all measures.

## Results

Bisegmental posturography findings (Table 3) showed significant postural differences between trials with eyes open and eyes closed in patients and healthy subjects. In healthy subjects, eye closure increased sway velocity and reduced MDD as postural control increased the weight of ankle in the balance between postural strategies adopted. In patients with CIDP, eye closure increased sway velocity and amplitude but left high MDD (hip strategy) unchanged.

**Table 3:** Platformless bisegmental posturography data in healthy subjects and patients with chronic inflammatory demyelinating poliradiculoneuropathy (CIDP).

	Healthy Subjects		Patients with CIDP					
	Eyes open	Eyes closed	Eyes open	Eyes Closed	† p <	‡ p <	§ p <	|| p <
**MDD**	0.36±0.01 (0.24–0.59)	0.27±0.01 (0.22–0.38)	0.45±0.02 (0.33–0.59	0.40±0.01 (0.33–0.52)	0.0001	NS	0.05	0.0000
**Mean Velocity T1**	5.55±0.23 (3.90–8.02)	9.37±0.45 (5.15–14.7)	9.53±1.21 (4.30–20.57)	22.71±3.58 (6.70–47.84)	0.00001	0.01	0.001	0.001
**Mean Velocity T2**	3.31±0.15 (1.95–4.99)	5.25±0.28 (2.72–8.19)	5.23±0.88 (2.62–13.78)	12.64±2.13 (3.82–30.60)	0.00001	0.01	0.01	0.01
**Mean Amplitude T1**	8.30±0.61 (4.72–15.14)	10.05±0.88 (4.95–23.7)	14.20±1.64 (7.19–27.23)	22.46±3.47 (8.05–51.62)	0.05	0.05	0.01	0.001
**Mean Amplitude T2**	5.25±0.39 (2.55–9.63)	5.95±0.56 (2.32–16.21)	8.69±0.96 (4.61–16.34)	11.52±1.81 (7.76–31.59)	NS	0.01	0.05	0.0000

^†^ = Wilcoxon test between eyes open and eyes closed condition in healthy subjects

^‡^ = Wilcoxon test between eyes open and eyes closed condition in CIDP patients

^§^ = Kruscal-Wallis Test between healthy subjects and CIDP patients, open eyes condition variables

^||^ = Kruscal-Wallis Test Test between healthy subjects and CIDP, open eyes condition variables

Values represent medians±standard errors and (min-Max).

Nearly all the postural variables studied differed significantly between healthy subjects and patients with CIDP. For example, in the eyes open and eyes closed conditions, mean MV at T1 and T2 was higher in patients than in healthy subjects. Similarly, MA was larger in T1 and T2 eyes open, and T1 and T2 eyes closed in patients than in controls. The MDD between T1 and T2 was higher in patients with CIDP than in healthy subjects.

A correlation was found between NDS sub-scores and some postural variables. In both the eyes open condition (r = 0.82, p<0.00), and the eyes closed condition (r = 0.79 p> 0.01) the MDD variable correlated with NDS-MD sub-score. Further analysis showed that NDS-MD sub-score also correlated with cMAP amplitudes of peroneal (r = -0.92, p<0.00) and tibial nerve (r = -0.83, p<0.00). Amplitude of peroneal and tibial nerve cMAPs also correlated with MDD in both the eyes open condition (peroneal nerve r = -0.87, p<0.00, tibial nerve r = -0.88, p<0.00), and the eyes closed condition (peroneal nerve r = -0.73, p<0.01, tibial nerve r = -0.73, p<0.00).

In the patients group, we found no correlation between age and postural variables, nor between age and disability score.

## Discussion

**Fig 1:**
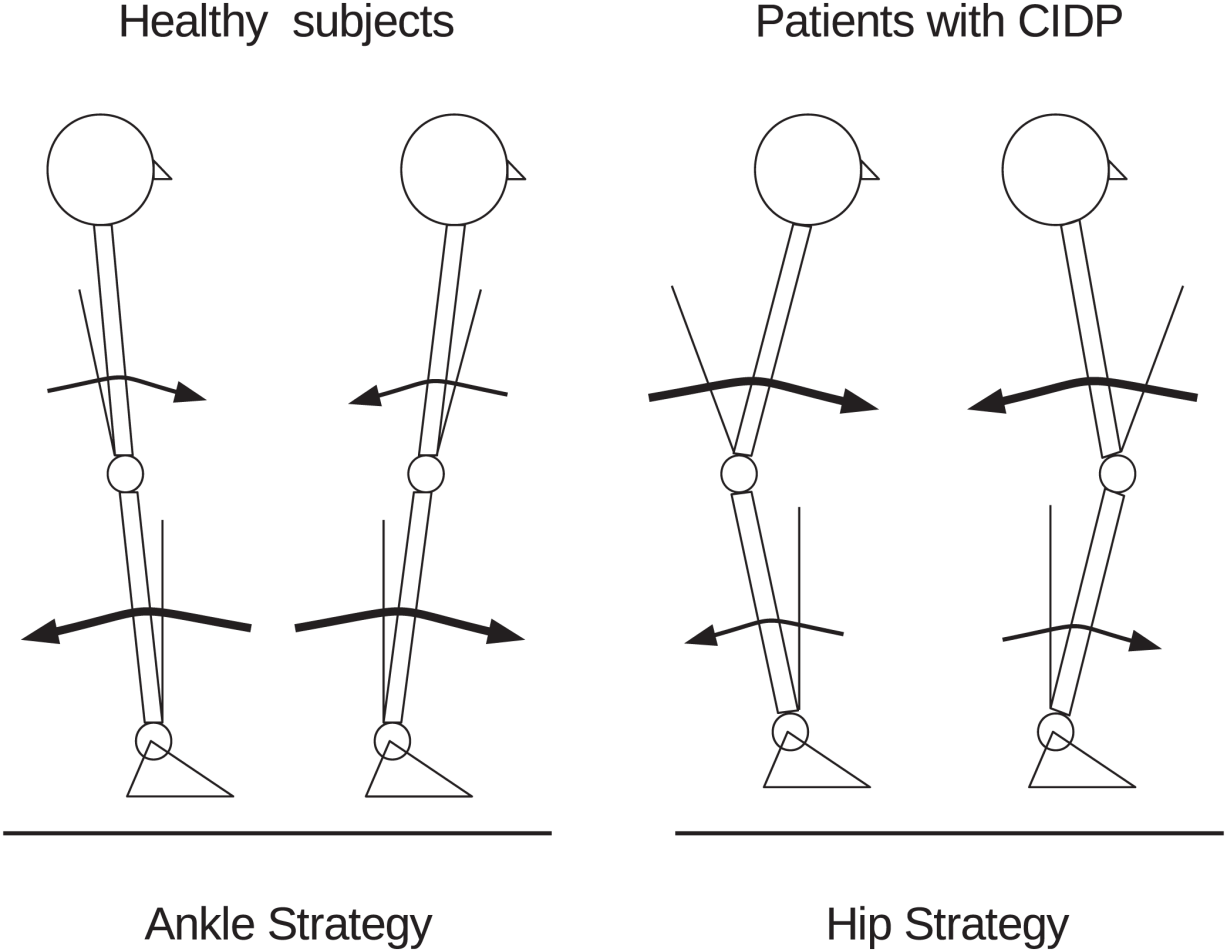
Balance between the postural strategies adopted to maintain stance in normal subjects and patients. Patients with chronic inflammatory demyelinating polyradiculoneuropathy overweigh hip strategy in the balance of postural strategies adopted during upright quiet stance.

Our clinical and posturographic study with the bisegmental posturography technique showed that patients with impaired somatosensory input and motor function related to CIDP at distal and proximal lower limbs prevalently maintain stance control under static conditions with a model based on a double-link inverted pendulum that activates hip strategy more than healthy subjects do (Fig 1). Conversely, healthy subjects prevalently keep the body upright with a single-link inverted pendulum that activates predominantly an ankle strategy. With the eyes closed, when the principal feedback sources are vestibular information and somatosensory input, whereas healthy subjects maintain the upright stance by accentuating their predominant reliance on a single-link inverted pendulum model, patients with CIDP are less efficient in increasing the activation of an ankle strategy, and continue using a double-link inverted pendulum model (i.e. a segmented body) that overweighs the activation of a hip strategy.

The ankle and hip strategies may be viewed as simultaneously co-existing excitable modes, both always present with varying amounts of power, one predominating on the other depending upon the characteristics of the available sensory information, biomechanical, environmental, and task constraints [10]. Owing to impaired distal nerve conduction, patients with CIDP cannot use sensory information from the ankle properly and have impaired motor function so they compensate postural sway by balancing their body with increasing the activation of hip strategy. Since not only CIDP induces dysfunction of distal but also proximal muscle districts, the new balance of strategies activated proves only partially efficient, because in the eyes-closed condition, although patients overweighed hip strategy (as shown by the not significant MDD change), they increased both the amplitude and the speed of postural sway.

Our detailed bisegmental posturographic findings in patients with CIDP agree with previous reports describing abnormal postural sway in patients with diabetic or hereditary peripheral polyneuropathy studied by standard posturography [27–31].

Reasonably, changes in vestibular input are unlikely to explain our findings because—apart from a report in a single patient [41]—CIDP spares the vestibular system. In addition, a previous study from our group showed that CIDP patients in the eyes closed condition activate compensatory strategies for proprioceptive afferents and visual deprivation by increasing vestibule-spinal excitability [42]. Changes in postural strategy in the patients studied here, rather depended mainly on slowed or scrambled sensory input (more evident with no vision), and motor impairment [16, 43].

Altered nerve conduction related to CIDP induces somatosensory impairment causing inappropriate signals from muscle and cutaneous sensory organs [13]. Accordingly, after normal and enhanced ankle input perturbations, stretch reflexes were absent in ankle and knee joint muscles in a patient with total proprioceptive loss in the legs [44]. Our patients’ difficulty in re-balancing postural strategies when studied without visual input, and preferences for a balance overweighting hip strategy with and without visual input receives support from a study describing diminished and delayed (by 45 ms) correcting responses in total proprioceptive loss in the legs [44]. The hip-predominant balance between strategies adopted, likely reflects how CIDP prevents patients from using somatosensory input generated by foot flexion, so that they can no longer stand upright and need to activate the hip muscles to move the trunk and maintain balance [16]. By increasing the flexibility at the head-hip-ankle axis, the hip strategy implies body segmentation. On the sensory side of the control process, segmentation improves detection of sway and activation of postural reflexes, because movements of the upper segments occur simultaneously with rotation of the ankles.

Predominant hip weighting in the balance between strategies adopted for postural correction during quiet stance in CIDP patients, may also reflect motor impairment in general, and unequal dysfunction of proximal and distal muscles in particular, as the correlation between postural variables and distal muscle component of NDS suggests. The central nervous system sums the individual sensory error signals and as a function of this summed signal generates an appropriate corrective torque signal [15, 45]. Lower-limb weakness causes instability because, although subjects can detect their sway, they cannot generate adequate stabilizing torques about the ankles to correct it [46–47]. Patients with mild diabetic neuropathy have difficulty in rapidly developing torque about the ankle from a lateral perturbation, possibly owing to a deficiency in distal motor function [21]. Similarly, our patients compensated postural sway by balancing their body increasing the activation at hip joint, as shown by their high MDD values, also owing to their predominantly distal weakness. Indeed, in the eyes-closed condition they proved less efficient than controls in increasing ankle strategy as normal subjects did. Since closing the eyes does not affect the contractile state (strength and rate of force development) of the leg muscles, whereas it affects predominantly the reliance on proprioceptive sensory input from the legs, CIDP patients maintained their ongoing balance of active postural strategies in the absence of visual input also owing to their distal muscle weakness. This interpretation is in line with observations that weaker subjects sway significantly more than stronger subjects when their sensory input is matched [23].

In patients, the MDD calculated during the eyes-closed condition was similar to that calculated in healthy subjects in the eyes-opened condition, i.e. a value we referred to as a “predominant ankle strategy”. However, when patients closed the eyes their MDD value did not change significantly from that calculated with the eyes opened, as it happened in controls. This apparent paradox resolves by contextualizing that ankle and hip strategies are not extremes along a continuum of mixed strategies to maintain upright stance, but they always co-exist with different relative intensities, reasonably dictated by biomechanical, environmental, and task constraints [10].

Clinical examination revealed that patients exhibited predominant motor component with a proximal to distal positive gradient. That the relative sparing of power in proximal muscle groups did not prevent CIDP patients from a partially inefficient re-balancing of postural strategies confirms a primary role of muscle weakness in determining their postural findings. This interpretation is supported by the correlation found between postural, clinical and neurographic variables (i.e., the greater the CIDP-related distal motor impairment, the greater the neurographic motor abnormality, and ankle-hip-head axis flexibility). Even in normal subjects, frontal plane hip strength proved a single best predictor of unipedal stance time and appeared to compensate for less precise ankle proprioceptor thresholds [48].

A limitation of the present study may be the position chosen for the top electromagnetic tracker, placed on the back of the head at inion. This prevented the link between the head and hip trackers to record the relative motion between the head and the trunk, and therefore to interpret the movements of the neck with respect to the trunk as wrongly reflecting a double-link inverted pendulum. Although we acknowledge that positioning the top tracker at the shoulder or the C7 spinous process would be advisable, we reasoned that during quiet stance head-trunk movements are negligible. Another limitation is that the study has been conducted on a small sample of highly clinically variable patients, thus precluding an adequate multivariate analysis. In addition, clinical variability may be at the origin of reduced changes in MDD in patients with the largest destabilizing responses when closing their eyes. These individuals had impaired somato-sensation and therefore were over-reliant on vision to control posture [49]. When vision was eliminated, their vestibular input might be insufficient to control posture, and they exhibited abnormally large sways. Reasonably, concurrent muscles weakness [20–21] prevented them from significantly changing the balance between strategies adopted to control posture as showed by their minimal MMD variation [50].

## Conclusions

We conclude that motor impairment together with slowed and scrambled proprioceptive afferent information related to CIDP cause our patients to overweigh hip strategy in their balance of postural strategies adopted during upright quiet stance. When patients with CIDP undergo posturography with the eyes closed they prove less efficient in increasing the relative activation of the ankle strategy as healthy people can, and they continue using a double-link inverted pendulum model that overweighs the activation of a hip strategy. Our findings show that in patients with muscle weakness even increasingly important multiple sensory inputs do not warrant stability. Future research is warranted to investigate the further correlations between postural variables and sensory-motor function.

## Supporting Information

S1 File Clinical, neurophysiological and posturographic measures in healthy subjects and patients. (XLS)
